# Pharmaceutical and pharmacokinetic evaluation of a newly formulated multiparticulate matrix of levodopa and carbidopa

**DOI:** 10.5599/admet.1474

**Published:** 2022-10-15

**Authors:** Emelia Priscilla Imbeah, Ofosua Adi-Dako, Benoit Banga N’guessan, Kennedy Kwami Edem Kukuia, Benedicta Obenewaa Dankyi, Ismaila Adams, Ebenezer Ofori-Attah, Regina Appiah-Opong, Seth Kwabena Amponsah

**Affiliations:** 1 Department of Medical Pharmacology, University of Ghana Medical School, Accra, Ghana; 2 Department of Pharmaceutics and Microbiology, School of Pharmacy, University of Ghana, Accra, Ghana; 3 Department of Pharmacology and Toxicology, School of Pharmacy, University of Ghana, Accra, Ghana; 4 Department of Clinical Pathology, Noguchi Memorial Institute for Medical Research, University of Ghana, Accra, Ghana

**Keywords:** Formulation, Parkinson’s disease, Management, Pectin, Pharmacokinetics, Chitosan

## Abstract

Levodopa is routinely co-administered with carbidopa in the management of Parkinson’s disease. Although the aforementioned combination therapy is effective, there may be fluctuating plasma levels of levodopa after oral administration. We formulated and evaluated the kinetic characteristics of the chitosan-pectin-based multiparticulate matrix of levodopa and carbidopa. Pectin was extracted from the cocoa husk, and the chitosan-pectin-based matrix was prepared by wet granulation. Formulations were evaluated for drug-excipient compatibility, drug content, precompression properties and in vitro release. For pharmacokinetic evaluation, rats were put into groups and administered either chitosan-pectin based matrix of levodopa/carbidopa, Sinemet^®^ CR or levodopa/carbidopa immediate release powder. Rats were administered the different formulations of levodopa/carbidopa (20/5 mg/kg) per os every 12 hours. The pharmacokinetic parameters of levodopa were estimated for the various treatment groups. The percentage content of levodopa and carbidopa in the various formulations was within the acceptance criteria. The AUC_0-24_ for levodopa/carbidopa multiparticulate matrix (Formulation 3: 484.98 ± 18.70 μg.hr/mL); Formulation 4: 535.60 ± 33.04 μg.hr/mL), and C_max_ (Formulation 3: 36.28 ± 1.52 μg/mL; Formulation 4: 34.80 ± 2.19 μg/mL) were higher than Sinemet^®^ CR (AUC_0-24_ 262.84 ± 16.73 μg.hr/mL and C_max_ 30.62 ± 3.37 μg/mL). The *t*_1/2_ of the new formulation was longer compared to Sinemet^®^ CR.

## Introduction

Parkinson’s disease (PD) is a debilitating disorder that affects the skeletal muscle system [1]. Some cardinal symptoms associated with PD include muscle rigidity, slow body movements, difficulty standing and tremor [2]. There could also be non-motor symptoms associated with PD, including sensory abnormalities, autonomic dysfunction, and dementia. Estimates suggest that more than 6 million people worldwide are affected by PD [3, 4].

A biological precursor of dopamine, levodopa, is the current mainstay in the management of PD. When levodopa is administered via the oral route, it is absorbed into the circulation and distributed to the brain. After levodopa crosses the blood-brain barrier, it is converted to dopamine, a process catalyzed by dopa decarboxylases. This leads to an increase in dopamine levels in the depleted striatum [2]. Orally administered levodopa exhibits low bioavailability (about 30 %); because of extensive metabolism by decarboxylases in peripheral circulation [5]. Furthermore, the systemic conversion of levodopa to dopamine often leads to unwanted side effects [6]. To reduce the metabolism of levodopa in the periphery, it is routinely co-administered with dopa decarboxylase inhibitors such as carbidopa. Although effective, a number of patients develop motor complications as treatment progresses with levodopa and carbidopa [6]. Reports suggest that these drawbacks result from fluctuations in the plasma concentration of levodopa [7].

Constant dopamine levels in the central nervous system could lower or prevent the emergence of motor fluctuations and dyskinesia in PD patients. Therefore, a number of studies have focused on the development of improved delivery systems for levodopa and other antiparkinson drugs. Most of these delivery systems aim to improve bioavailability and minimize unwanted motor complications of levodopa [8–10]. Over the last few decades, natural and biocompatible polymer matrices have been used as carriers for sustained drug release [11,12]. Chitosan and pectin are biopolymers that are readily available, eco-friendly, immunocompatible, and non-toxic. Chitosan and pectin are less expensive compared to synthetic polymers such as polylactic acid, and polyglycolic acid, among others. Cocoa pod husk (CPH) pectin is an anionic polysaccharide often used as a multifunctional pharmaceutical excipient [13]. CPH pectin is known to be non-toxic and swells at varying extents [14,15]. The swelling characteristics of CPH pectin make it a suitable binder or matrix in controlled-release formulations [15].

Indeed there have been previous studies that have assessed chitosan-based levodopa carbidopa formulations using different routes of administration; intraduodenal infusion, nasal and oral [16,17]. However, there is a paucity of data on the oral chitosan-pectin-based multiparticulate matrix of levodopa and carbidopa. For an oral formulation, chitosan has several positively charged groups which readily interact with the negatively charged mucous membranes of the gastrointestinal tract, thereby increasing adhesion. For our formulation, the addition of pectin (anionic polysaccharide) causes chitosan and pectin to interact and form a polyelectrolyte complex. Studies have shown that polyelectrolyte complexes have the ability to encapsulate drugs in a polymeric matrix at the molecular level, thereby enhancing the physicochemical and pharmacokinetic characteristics of drugs. The current study adopts a simple and cost-effective formulation approach, using optimized chitosan-pectin-based formulations to be administered via the oral route. The formulation was evaluated for its pharmaceutical and *in vivo* pharmacokinetic characteristics.

## Experimental

### Materials

Levodopa and carbidopa powders, low molecular weight chitosan and microcrystalline cellulose, were purchased from Sigma-Aldrich (St Louis, Missouri, USA). Sinemet^®^ CR tablet 100/25mg (Merck Sharp & Dohme Limited, Hertfordshire, UK) was purchased from Medimart Pharmacy, Accra, Ghana. All solvents used for chromatography were of high-performance liquid chromatography (HPLC) purity. All other reagents used were purchased from approved suppliers and were of analytical grade.

### Extraction of pectin

Cocoa pods were harvested from *Theobroma cacao* L. trees. To avoid pigmentation, the pulp and seeds were removed. Afterwards, the pod husks were peeled and blended. Extraction of pectin from fresh CPH was done in a water bath (50 °C) and precipitated with ethanol, according to a procedure previously described by Adi-Dako et al. [15], with minor modifications. Pectin yield was then determined, and samples were covered with aluminium foil and kept in a desiccant.

### Characterization of extracted hot water soluble CPH pectin by Fourier transform infrared spectroscopy (FTIR)

FTIR spectrum of the CPH pectin was done using a Perkin Elmer UATR-II FTIR spectrometer (Perkin Elmer Ltd, Beaconsfield, UK). The pectin was placed onto the attenuated total reflectance (ATR) crystal surface and the spectrum was determined over a wavelength range of 4000-500 cm^–1^ and a resolution of 1.0 cm^1^.

### Formulation of multiparticulate matrix of levodopa and carbidopa

Briefly, chitosan was weighed and dissolved in 1 % glacial acetic acid. The mixed powder of levodopa and carbidopa was triturated with the chitosan solution until a uniform mixture was obtained. An adequate volume of hot water was added to the CPH pectin with stirring. The mixture was dried at 30 °C for 8 hr. Afterwards, the dried mass was milled, passed through a sieve (No. 40), weighed and stored in glass containers at room temperature. The formulation details of the various multiparticulate matrices of levodopa/carbidopa (F1, F2, F3, F4 and F5) are summarized in Table 1.

### Drug-excipient compatibility

A Perkin Elmer UATR-II FTIR spectrophotometer (Perkin Elmer Ltd, Beaconsfield, UK) operating on Diamond ATR was used to evaluate drug-excipient compatibility. The spectra of levodopa, carbidopa, chitosan, pectin and the chitosan-pectin-based matrix were measured over a wavelength range of 4000-500 cm^–1^ and at a resolution of 1.0 cm^1^. Spectra for levodopa, carbidopa, chitosan, pectin and the chitosan-pectin-based matrix of levodopa/carbidopa were superimposed.

### Flow properties of chitosan-pectin-based multiparticulate matrix

Briefly, 3 g of the various formulations were weighed into a 100 ml graduated measuring cylinder and the volume occupied was noted as V1. The cylinder was then tapped until the powders were consolidated. The volume obtained after tapping was designated V2. The tapped and bulk densities, Carr’s index and Hausner ratio, were estimated based on the formulae:



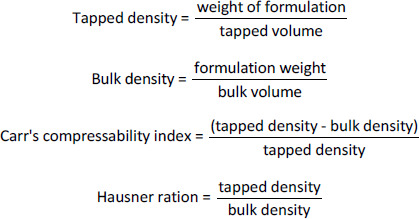



In order to determine the angle of repose, 5 g of the formulations were weighed, transferred into a clamped funnel (with the tip about 10 cm from a plain paper placed below it) and allowed to flow unto the surface of the paper freely. The height (*H*) of the cone formed was noted. A circle was drawn around the base of the cone and the radius (*R*) was determined. The repose angle, *Θ*, was estimated as follows:



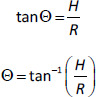



### Drug content

Levodopa and carbidopa in the various formulations were determined according to a validated HPLC method previously described by Shohreh et al. [18], with minor modifications. The chromatographic procedure was carried out using Agilent Technologies system 1100 (Santa Clara, CA, USA) equipped with a UV/visible detector (detection wavelength of 280nm). A stainless-steel column with stationary phase Tskgel ODS C18 maintained at 30 °C was used. The mobile phase comprised 10 mM phosphate buffer (pH of 4.0) and methanol (90:10 v/v). The flow rate of the mobile phase was maintained at 0.6 ml/min with a run time of 10 min. The injection volume was set to 20 μl. All solvents used were of HPLC grade and were filtered with 0.45 μm filters prior to use.

### Preparation of stock and working solutions

Stock standard solutions (1 mg/ml) of levodopa and carbidopa were prepared fresh daily in 1000 μL distilled water separately. The respective solutions were vortex-mixed for 10 min and sonicated for 20 min to completely dissolve the drugs. A working standard solutions of 200 μg/ml of each stock were prepared. Two-fold serial dilutions of each working standard solutions were prepared to give eight standard solutions with concentrations 100, 50, 25, 12.5, 6.25, 3.125, 1.56 and 0.78 μg/mL. The same volume of standard solutions of levodopa and carbidopa were mixed, giving standard working solutions 100, 50, 25, 12.5, 6.25, 3.125, 1.56, 0.78 and 0.39 μg/mL. Twenty microliters (20 μL) of the standard working solutions were injected into the HPLC system. The retention times and peak areas for levodopa and carbidopa were determined and calibration curves were obtained. Levodopa and carbidopa showed good resolution and retention times (3.7 and 5.5 min, respectively).

### Validation of the Method

The analytical method was validated according to the International Conference on Harmonization (ICH) guidelines.

#### Linearity

The linearity was evaluated by analyzing different concentrations of the standard solutions. The calibration curve was constructed for levodopa and carbidopa by plotting the average peak area against concentration, and a regression equation was found from the plot. The slope of the regression line and values of the correlation coefficient (*R*^2^) for each standard curve were obtained by using MS Excel software.

#### Determination of LOQ and LOD

Limits of detection (LOD) and limits of quantification (LOQ) were calculated for each standard solution in triplicates. The mean of the slope (S) and standard deviation of the response (σ) were calculated from the standard curve of three replicates. LOD and LOQ were calculated with the following equations:



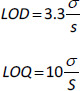



#### Precision

The precision of the method was tested by injecting a standard solution of Levodopa and Carbidopa (12.5 μg/mL) six times. Peak areas were determined, compared and expressed as percentage relative standard deviation (% RSD):



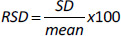



#### Selectivity

Selectivity was evaluated by processing and analyzing drug-free samples to ensure the absence of compounds with the same retention times at the analytes of interest. No peaks were observed at the retention times of levodopa and carbidopa.

#### Robustness

The robustness of the procedure was demonstrated by intentionally modifying the chromatographic conditions. The mobile phase flow rate was altered from 0.6 to 0.5 mL/min and from 0.6 to 0.7 mL/min. The column temperature was varied from 30 °C to 35 °C, as well as the wavelength from 280 to 282 nm. The percentage recovery of robustness testing under the altered conditions was calculated in all cases.

### Content analysis

The actual drug content of the chitosan-pectin-based multiparticulate matrix was determined by weighing and dissolving 1 mg of the formulation (in triplicate) in 1 ml of distilled water. This was then vortex-mixed for 10 min, and sonicated for 10 min. Afterwards, the mixture was centrifuged at 10,000 rpm for 10 min. A volume of 500 μL of the supernatant was pipetted into the HPLC auto-sampler and analysed by HPLC (as previously described).

### In vitro drug release

Drug dissolution was conducted in phosphate buffer (pH 6.8) to simulate the gastrointestinal environment, as previously described [10,19]. Samples of the various chitosan-pectin-based formulations (F1, F2, F3, F4 and F5) were tested *in vitro*. Samples (5 ml) were drawn from baskets of USP dissolution apparatus at the following time points: 0, 0.5, 1, 2, 4, 8, 12 and 24 hr, for analysis. These were immediately replaced with an equal volume of fresh phosphate buffer (37 °C). The release of levodopa and carbidopa was determined by assaying levels using HPLC, as previously described by Shohreh et al. [18]. Triplicate measurements were performed.

*In vitro* drug release was also done in a phosphate buffer of pH 4.5 (for optimized formulations F3 and F4 only) to mimic the duodenum-jejunal environment where absorption of levodopa occurs [20,21] and to observe the behavior of the chitosan-pectin matrix in this media.

### Pharmacokinetic evaluation of new formulation

Sprague Dawley (SD) rats that were about seven weeks old and weighing between 180-200 g were housed in an approved animal house. Animals were kept at optimal laboratory conditions, which consisted of 12-hour lightening, laboratory temperature of 25 ± 1 °C, and humidity of 60-70 %. SD rats were made to acclimatise to this laboratory condition for two weeks. Guidelines for animal use and care[22], were adhered to throughout the experiment.

Animals were made to fast overnight and rats in Group 1 were administered an optimized chitosan-pectin-based matrix of levodopa and carbidopa (F3), and rats in Group 2 were given F4. These two formulations showed better pharmaceutical and *in vitro* kinetic characteristics. Group 3 rats received Sinemet^®^ CR (Merck Sharp & Dohme Limited, Hertfordshire UK). Group 4 rats were given immediate-release powders of levodopa and carbidopa. Animals in each group were given a dose of (20/5 mg/kg) of levodopa and carbidopa *per os* every 12 hours. The multiparticulate matrix formulations, Sinemet^®^ CR and the immediate release powders were suspended in water before administration (via oral gavage). After the third dose, tail vein samples were taken at 0.25, 0.5, 1, 2, 4, 8, 12 and 24 hr into ethylenediaminetetraacetic acid (EDTA) tubes. In order to minimize levodopa in blood samples from undergoing oxidation, 25 % sodium metabisulfite in water was prepared and added to the blood in a ratio of 1:10 (v/v). Immediately, samples were centrifuged at 4,500 rpm for 10 min to obtain plasma. Levodopa in plasma was assayed using reverse-phase HPLC, as previously described by Kim et al. [23]. A stock of the internal standard solution was prepared by dissolving 20 mg methyldopa in 15 mL deionized water to obtain a concentration of 1.3 mg/mL. Levodopa was extracted from plasma using protein precipitation with perchloric acid. To 100 μL of rat plasma, 50 μL of 0.1 M perchloric acid was added. The mixture was vortexed for 2 min. Afterwards, centrifugation was done at 10,000 rpm and the supernatant analysed by HPLC. The HPLC method showed good linearity (0.00 – 25.00 μg/mL) with a correlation coefficient (R^2^) of 0.9997 for the calibration curve of levodopa.

### Ethical considerations

The Ethics and Protocol Review Committee, College of Health Sciences, University of Ghana, approved this study. The Protocol Identification Number is CHS-Et/M.5 – 4.4/2020 – 2021.

### Data analysis

Data were expressed as mean with standard deviation. The pharmacokinetic parameters of levodopa in the treatment groups were determined by non-compartmental analysis. Inferential statistics; one-way analysis of variance (ANOVA) followed by Tukey’s *post hoc* multiple comparison test were used to compare pharmacokinetic parameters. Plots were done in GraphPad Prism 7.0 (San Diego, California) and Excel 2013. *P* value less than 0.05 was deemed statistically significant.

## Results and Discussion

### FTIR analysis of CPH pectin

**Figure 1.**
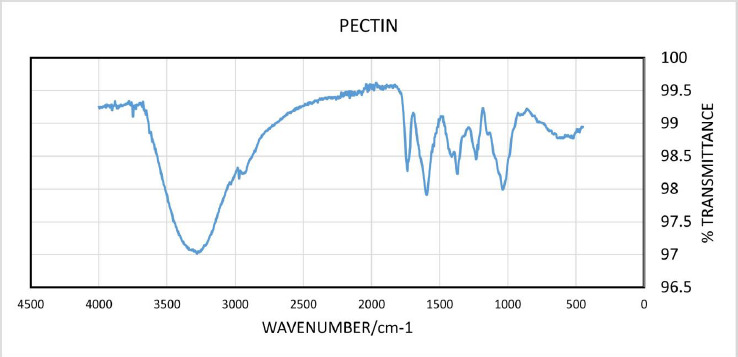
FTIR spectrum of CPH pectin

The yield of the hot water-soluble pectin from CPH was 7.91 %. Infra-red spectrum of CPH pectin (Figure 1) showed broad absorption bands at 3280 cm^-1^, 2932 cm^-1^, 1735 cm^-1^ and 1596 cm^-1^. Other absorption bands were observed between and 1500 cm^-1^ and 1428 cm^–1^.

### FTIR – drug-excipient compatibility study

**Figure 2.**
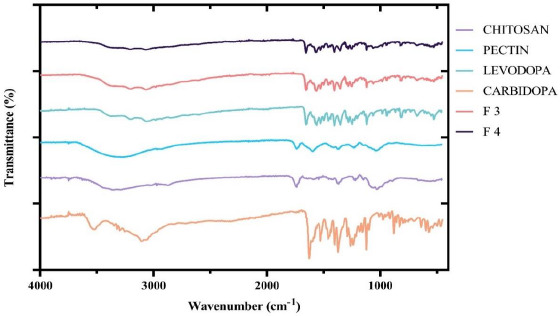
FTIR Spectra of levodopa, carbidopa, CPH pectin, chitosan and optimized formulations (F3 and F4)

The FTIR spectra of pure levodopa, pure carbidopa, chitosan, CPH pectin and the two optimized formulations, F3 and F4, were obtained and superimposed. As shown in Figure 2, the characteristic peaks of levodopa and carbidopa were retained in the chitosan-pectin-based formulations (optimized formulations F3 and F4).

### Flow and precompression parameters

Carr’s compressibility index, bulk and tapped densities, Hausner ratio and angle of repose of the various chitosan-pectin-based formulations were determined. The results are shown in Table 2. All the formulations had satisfactory flow properties.

### Drug content and loading efficiency

The content (%) of levodopa and carbidopa in the various formulations is shown in Table 4. The content of levodopa and carbidopa in formulation 5 (F5) was above the acceptable criteria. Acceptance criteria: levodopa/carbidopa extended-release formulations should contain not less than 90.0 % and not more than 110.0 % of the stated amount of levodopa and carbidopa. Additionally, the loading efficiencies of the various formulations are shown in Table 4.

### In vitro drug release

**Figure 3.**
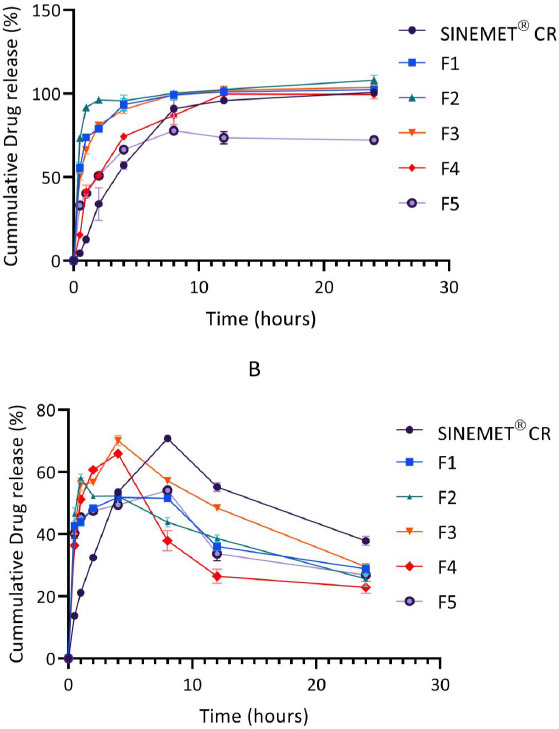
*In vitro* release of levodopa (A) and carbidopa (B) from the various formulated multiparticulate matrices in phosphate buffered saline (pH = 6.8) at 37 °C (n=3). Error bars indicate SD.

**Figure 4.**
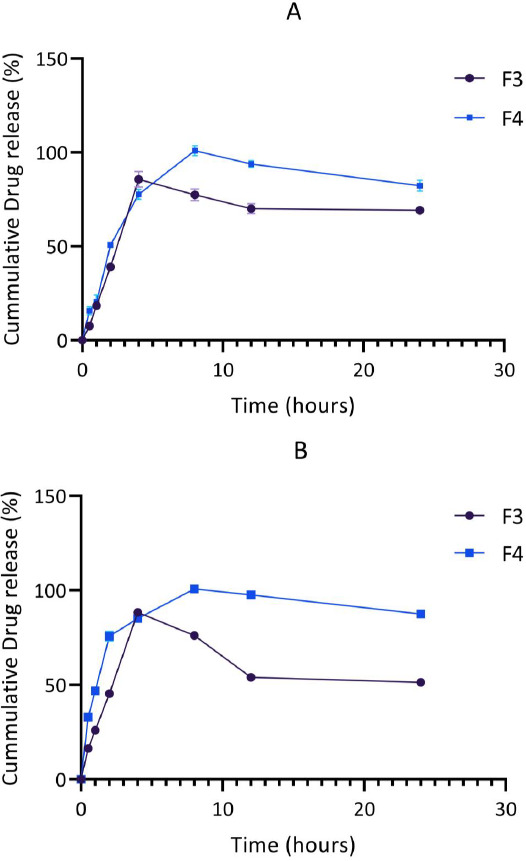
*In vitro* release of levodopa (A) and carbidopa (B) from the optimized formulations F3 and F4 in phosphate buffered saline (pH = 4.5) at 37 °C (n=3). Error bars indicate SD.

Over a 24 hr period, the release of levodopa and carbidopa are shown in Figure 3 and Figure 4. In general, levodopa release was found to increase steadily. The release of carbidopa, however, declined sharply in phosphate buffer (pH 6.8) after a 4 hr period for all formulations.

### Concentration-time curves

**Figure 5.**
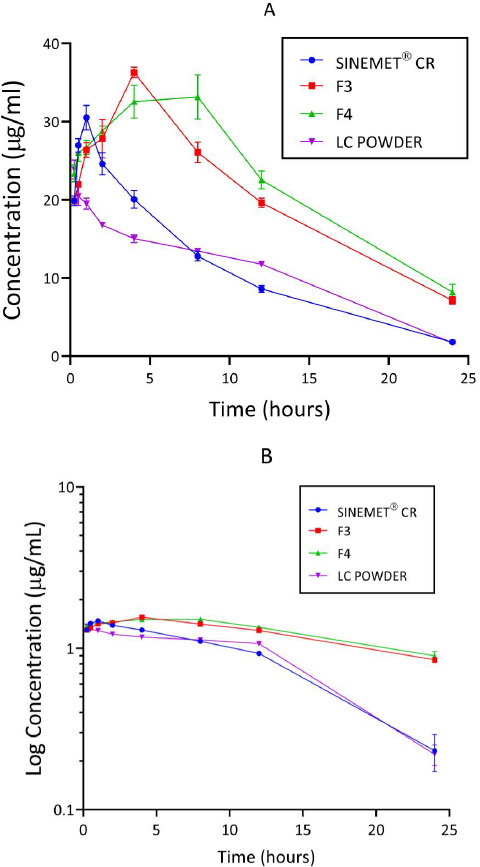
Plasma concentration-time (A) and log concentration-time (B) curves of levodopa for the 4 treatment groups (n = 5) following administration of respective formulations administered at 20/5 mg/kg. Sinemet^®^ CR = a controlled release formulation of levodopa/carbidopa. F3 = Formulation 3, F4 = Formulation 4, LC = levodopa plus carbidopa powder. Error bars indicate SD.

Concentration-time curves of levodopa for the treatment groups are shown in Figure 5 (A and B). Peak concentration (*C*_max_) and time to reach the peak (*T*_max_) were higher for the groups administered a chitosan-pectin-based matrix of levodopa/carbidopa. Rats administered pure levodopa/carbidopa had the lowest C_max_.

### Pharmacokinetic parameters

The pharmacokinetic parameters of levodopa derived from concentration-time and log concentration-time plots are shown in Table 4. One-way ANOVA performed on *T*_max_, *C*_max_, area under the concentration-time curve (AUC), elimination rate constant (*K*_e_) and half-life (*t*_1/2_) varied significantly (p < 0.05) between the four treatment groups. *C*_max_ of levodopa was found to be higher for formulations F3 and F4. AUC_0-24_ and AUC_0-∞_ of levodopa for formulations F3 and F4 were found to be 2-fold greater than the other formulations. Additionally, the *t*_1/2_ for formulations F3 and F4 were relatively longer compared to Sinemet^®^ CR. A comparison of the AUC_0-∞_ and AUC_0-24hr_ between F3 and Sinemet^®^ CR and between F4 and Sinemet^®^ CR were all found to differ significantly (p < 0.0001). A comparison between the *t*_1/2_ of F3 and Sinemet^®^ CR showed that the difference observed was statistically significant (p <0.0001). The difference between the *t*_1/2_ of Sinemet^®^ CR and F4 was also found to be significant (p <0.0001).

Cocoa pod husk (CPH) is an environmentally and economically friendly means of managing CPH waste after harvesting the beans from the pods. In the current study, the yield of hot water-soluble pectin from CPH was 7.91 %. Previous studies have reported extraction yields of 23.3 % and 6.5 % for hot water-soluble pectin [13,24,25]. The extraction yields of CPH pectin with hot water (pH 7.0) have been shown to depend largely on factors such as the origin of cocoa pods, extraction time, as well as the pre-treatment methods employed [25,26].

Data obtained from FTIR spectroscopy of CPH pectin showed a broad absorption band at 3280 cm^-1^, which corresponds to hydroxyl (-OH) stretching. A band at 2932 cm^-1^ corresponds to tension in C-H due to the vibration of methyl ester groups. The sharp absorption bands at 1735 cm^–1^ and 1596 cm^-1^ show esterified and non-esterified carboxyl groups in the CPH pectin. Absorption signals between 1623 and 1428 cm^–1^ usually correspond to wavelength features of polygalacturonic acid [27,28]. Thus, the pectin extracted from CPH can be said to be rich in polygalacturonic acid [29].

Polymers such as alginate, dextran, pectin, guar gum and chitosan have been employed to control and sustain the release of drugs [30,31]. In this study, chitosan and CPH pectin were employed in the formulation of an oral multiparticulate matrix of levodopa and carbidopa. Oral multiparticulate matrix systems have been proven suitable for modified-release formulations. These granular matrix systems are also known to have predictable gastrointestinal transport and a low risk of dose dumping [32,33].

A number of methods, such as differential scanning calorimetry, isothermal stress testing and FTIR, have been employed in drug-excipient compatibility studies. Among these methods, FTIR was found to be useful in providing information on drug/excipient compatibility [34,35].

Drug-excipients compatibility test was done by FTIR in order to predict any potential chemical or physical interactions that could affect the quality, physicochemical properties and release of active pharmaceutical ingredients [36]. In the FTIR spectrum of levodopa, characteristic peaks appearing between 3500 cm^-1^ – 3200 cm^-1^ show O-H stretching. Bands between 3000 cm^-1^ – 2850 cm^-1^ are indicative of symmetric and asymmetric -C-H stretches. Secondary amine (-NH_2_) stretches were visible at 1560 cm^-1^, and a sharp peak at 1633 cm^-1^ corresponded to the presence of a carbonyl (C=O) [37]. FTIR spectrum of carbidopa showed visible absorption bands between 3500-3200 cm^-1^ and 3100-3000 cm^-1^ corresponding to O–H stretches and -C–H stretches, respectively. Carbonyl peak was visible at 1627 cm^-1^, NH_2_ (secondary amine) absorption band appeared at 1560 cm^-1^ and absorption bands appearing between 1450 cm^-1^ - 1400 cm^-1^ corresponded to phenyl group C=C vibrations [38]. The spectrum of chitosan showed characteristic absorption bands of C=O stretching and amidic N-H bending between 1700 cm^-1^ - 1650 cm^–1^ and 1500 cm^-1^ -1400 cm^–1^, respectively [39]. The spectrum of pectin showed characteristic peaks as previously described. FTIR results of optimized formulations (F3 and F4) showed similar peaks for the specific functional groups present in levodopa and carbidopa (shown in Figure 2). This finding suggests no structural change or interference to levodopa and carbidopa with excipients used. Previous reports by Bigucci et al. [39] and Gadalla et al. [40] also revealed that chitosan and pectin were compatible with vancomycin and progesterone, respectively.

In drug formulation, the ease of flow of granular powders is important. This is because free-flowing powders ensure reproducible dosator filling [13,41]. The flow and precompression parameters of the chitosan-pectin-based formulations studied were Carr’s index, Hausner ratio, and angle of repose. For the Hausner ratio, values close to 1.2 suggest free-flowing and less cohesive powders, while values greater than 1.6 indicate cohesive powders with poor flowability [42]. Carr’s compressibility index above 32 and angles of repose above 35° are indicative of powders with poor and unsatisfactory flow properties [43]. In this study, formulations F1, F2, F3, F4 and F5 were found to be less cohesive and had satisfactory flow properties.

According to the United States Pharmacopoeia [44], levodopa and carbidopa combination should contain not less than 90 % and not more than 110 % of the stated amount of levodopa and carbidopa. In the current study, the content of levodopa and carbidopa in all formulations with the exception of F5, were within the stated acceptance criteria.

The *in vitro* drug release in phosphate buffer was conducted to mimic the physiological gastrointestinal environment [10,19]. The release of the levodopa from the matrix was found to be controlled and sustained over 24 hr. As shown in Figure 3, levodopa was rapidly released from formulation F1 (only chitosan) and formulation F2 (100 mg chitosan and 50 mg pectin). Increasing the amount of pectin in F3 (containing 100 mg chitosan and 100 mg pectin) resulted in a reduced release rate of levodopa from the chitosan-pectin matrix. These results are consistent with those of El-Gibaly [44], who also reported a more delayed release of drug when the concentration of pectin in a matrix was increased.

The addition of hydroxyapatite and calcium chloride salts to the chitosan-pectin matrix as seen in F4 (100 mg chitosan, 100 mg pectin and 10 mg hydroxyapatite) and F5 (100 mg chitosan, 100 mg pectin and 50 mg CaCl_2_) further delayed the release of both levodopa and carbidopa from these formulations. Hydroxyapatite is a calcium-rich mineral. Calcium ions serve as cross-linking agents for pectin and earlier reports suggest that the binding of calcium ions with pectin delay drug release and increase the gel strength of polymer matrices [45–47]. Findings from the *in vitro* drug release profiles of F4 and F5 also suggested that calcium ions from CaCl_2_ provided better cross-linking with pectin than hydroxyapatite. The release profile of F5, was, however, unsatisfactory, as only 77.8 % of levodopa was released from the multiparticulate matrix.

In comparison with Sinemet^®^ CR, the release of levodopa from the new formulation was found to be biphasic, with an averagely of 50 % of the drug released in the first 1 hr (as against ~ 12 % for Sinemet^®^ CR) and the remaining drug released in a controlled fashion over the next 24 hr. In biphasic oral drug delivery systems, the immediate release component provides the loading dose while the sustained release portion maintains effective plasma drug concentration over time [46,48,49].

The release of carbidopa from the chitosan-pectin multiparticulate matrix and Sinemet^®^ CR increased gradually over 4 hr and 5 hr, respectively, and declined sharply beyond these time points. This finding was found to be consistent with earlier reports [19]. Carbidopa is reported to be unstable at pH 6.8 [21]. Hence, significant degradation might have occurred during the *in vitro* drug release studies (Figure 3B).

Based on the aforementioned content analysis and drug release profiles of the five chitosan-pectin-based formulations (F1, F2, F3, F4 and F5), formulations 3 and 4 were selected as optimized for further investigations.

The release patterns of levodopa and carbidopa in the optimized formulations (F3 and F4) were further investigated in phosphate buffer (pH 4.5), mimicking the conditions of the duodenum-jejunal region of the gastrointestinal tract where absorption of levodopa occurs. The release of levodopa and carbidopa was found to be controlled. In this medium, the maximum release of levodopa and carbidopa from F3 and F4 occurred after 4 hr and 8 hr, respectively.

In the current study, only the pharmacokinetic parameters of levodopa were considered; as seen in other studies [50,51]. Findings from the concentration-time plots of levodopa showed that higher peak levodopa plasma concentrations (*C*_max_) were achieved with F3 and F4 compared to other formulations. Previous studies showed similar data for carvedilol and anthracyanins when chitosan-pectin complexes were used [48,49]. Levels of levodopa in plasma have been shown to be directly correlated with the amount reaching the brain [52]; hence a higher *C*_max_ may result in an increase in the levels of levodopa in the brain. This may be advantageous in the management of PD.

In this study, *C*_max_ for F3 and F4 were reached after 4.0 (± 0.00) hr and 4.4 (± 2.19) hr, respectively. *C*_max_ for Sinemet and the levodopa/carbidopa powder were reached after 0.9 (± 0.22) hr and 0.4 (± 0.34) hr, respectively. Thus, the matrix formulations demonstrated a prolonged release of levodopa. Such prolonged-release formulations of levodopa/carbidopa are known to reduce the frequency of dosing and enhance patient compliance [53,54].

The AUC of the various formulations showed that F3 and F4 were greater, about twice that of the AUC of Sinemet^®^ CR (Table 5). AUCs from concentration-time curves reflect total drug exposure after drug administration [55]. The high AUC values of F3 and F4 could be attributed to the chitosan's outstanding mucoadhesive properties conferred on the matrix. Chitosan, a mucoadhesive polymer, is positively charged and readily interacts with mucous membranes of the gastrointestinal tract (negatively charged), thereby increasing adhesion and thus improving contact time for drug absorption [56]. Furthermore, chitosan has been shown to have permeation-enhancing properties [57,58]. Since AUC is a reliable measure of the bioavailability of a drug, it can be inferred that the chitosan-pectin-based matrix of levodopa/carbidopa had greater bioavailability than Sinemet^®^ CR (conventional controlled-release product on the market).

Furthermore, the current study showed that half-life was longest for F4 (8.84 ± 2.95 hr), followed by F3 (8.33 ± 0.01 hr) and Sinemet^®^ CR (5.34 ± 0.95 hr). Half-life has been shown to play a key role in determining the duration of action of a drug [55]. Data from this study suggests that it takes a relatively long time for levodopa within the chitosan-pectin-based matrix to be cleared from plasma. This property may be of great benefit in preventing the “on” and “off” phenomenon observed with the use of conventional levodopa/carbidopa formulations. Chitosan-pectin-based matrix provides a complex that controls and delays the release of drugs into plasma [59–62].

## Conclusions

The oral multiparticulate matrix of levodopa/carbidopa exhibited modest pharmacokinetic characteristics compared to the conventional controlled release formulation (Sinemet^®^ CR) and levodopa/carbidopa powder. The mucoadhesive nature of the matrix and sustained delivery of levodopa can be employed in overcoming the irregular gastric emptying and motor fluctuations (‘on’ and ‘off’ phenomenon) associated with oral administration of levodopa. Future studies should be conducted to evaluate the biodistribution and efficacy of this new formulation.

## Figures and Tables

**Table 1.** Composition of chitosan-pectin-based multiparticulate matrix of levodopa/carbidopa

Ingredient	F1 (mg)	F2 (mg)	F3 (mg)	F4 (mg)	F5 (mg)
Levodopa	100	100	100	100	100
Carbidopa	25	25	25	25	25
CS	100	100	100	100	100
CPH-pectin	-	50	100	100	100
HA	-	-	-	10	-
CaCl_2_	-	-	-	-	50
MCC	200	150	100	90	50
**Total**	425.0	425.0	425.0	425.0	425.0

*CS = chitosan, MCC = microcrystalline cellulose, HA = hydroxyapatite, CaCl_2_ = calcium chloride, F1 = formulation 1, F2 = formulation 2, F3 = formulation 3, F4 = formulation 4 and F5 = formulation 5*

**Table 2.** Flow properties of chitosan-pectin-ased formulations

Formulation	Bulk density (kg/m^3^)	Tapped density (kg/m^3^)	Hausner ratio	Carr's index	Angle of repose (°)
F1	566.04	697.67	1.23	18.87	27.15
F2	545.45	750.00	1.38	27.27	23.5
F3	612.24	714.29	1.17	14.29	24.64
F4	588.24	697.67	1.19	15.69	28.61
F5	517.24	666.67	1.29	22.41	24.44

Reference ranges for Hausner ratio, Carr’s index and angle of repose are: Carr’s index < 32 %, Hausner ratio < 1.5, Angle of repose < 35

**Table 3.** Validation parameters of the HPLC method quantification of levodopa and carbidopa

Validation Parameters	Results	
	Levodopa	Carbidopa
Retention time [Mean ± S.D. (*n*=7)]	3.70 ± 0.186	5.50 ± 0.0018
Linear range (μg/mL)	0.00– 25.00	0.00– 12.50
Correlation coefficient (R^2^)	0.9997	0.9994
Regression equation	y = 29.469x + 2.9818	y = 12.794x + 1.2489
Precision (*n*=6 % RSD)	0.6050	1.875
LOQ (μg/mL)	1.3405 ± 0.056	1.1355 ± 0.04
LOD (μg/mL)	0.6319 ± 0.0119	0.5353 ± 0.021
Wavelength λ (nm)	280	280
Robustness (Flowrate: 0.5 mL/min)	99.044 ± 0.085	98.140 ± 0.180
Robustness (Flowrate: 0.7 mL/min)	100.039 ± 0.067	100.933 ± 0.119
Robustness (Temperature 35 °C)	98.320 ± 0.085	98.015 ± 0.078
Robustness (wavelength 282 nm)	100.311 ± 0.087	101.558 ± 0.090

SD: Standard deviation, RSD: Relative standard deviation, LOQ: Limit of quantification, LOD: Limit of detection

**Table 4.** Drug content and loading efficiency of formulations

Formulation	Average % content ± STDEV		Loading efficiency (%) ± STDEV	
	Levodopa	Carbidopa	Levodopa	Carbidopa
F1	107.20 ± 10.56	104.73 ± 3.67	75.43 ± 6.52	63.43 ± 4.22
F2	91.25 ± 5.29	96.59 ± 3.93	81.23 ± 4.11	68.31 ± 6.69
F3	100.70 ± 0.12	99.03 ± 2.57	93.86 ± 3.26	85.23 ± 4.57
F4	93.51 ± 0.28	91.96 ± 1.82	89.31 ± 2.52	81.35 ± 1.77
F5	114.68 ± 5.26	114.73 ± 1.62	65.43 ± 6.68	55.91 ± 8.63

**Table 5.** Pharmacokinetic parameters of levodopa in the four treatment groups (± SEM)

PK parameter	Sinemet^®^ CR	F3	F4	LC	p-value
*T*_max_ (hr)	0.9 (0.22)	4 (0.00)	4.4 (2.19)	0.40 (0.34)	<0.0001
*C*_max_ (μg/ml)	30.62 (3.37)	36.28 (1.52)	34.80 (2.19)	24.00 (2.42)	<0.0001
AUC_0-24_ (μg.hr/ml)	262.84 (16.73)	484.98 (18.70)	535.60 (33.04)	252.39 (135.47)	<0.0001
AUC_0-∞_ (μg.hr/ml)	275.60 (16.89)	572.13 (36.46)	647.40 (83.55)	262.83 (10.35)	<0.0001
*K*_e_ (1/hr)	0.13 (0.03)	0.09 (0.02)	0.09 (0.03)	0.16 (0.01)	0.0003
*t*_1/2_ (hr)	5.34 (0.95)	8.33 (0.01)	8.84 (2.95)	4.27 (0.35)	<0.0001

Sinemet^®^ CR = a controlled release formulation of levodopa/carbidopa. F3 = Formulation 3, F4 = Formulation 4, LC = levodopa plus carbidopa powder
